# Short Time Correlation Analysis of Melt Pool Behavior in Laser Metal Deposition Using Coaxial Optical Monitoring

**DOI:** 10.3390/s21248402

**Published:** 2021-12-16

**Authors:** Yury N. Zavalov, Alexander V. Dubrov

**Affiliations:** Institute on Laser and Information Technologies—Branch of the Federal Scientific Research Centre “Crystallography and Photonics” of Russian Academy of Sciences, Svyatoozerskaya 1, 140700 Shatura, Moscow Region, Russia; zavalov@laser.ru

**Keywords:** laser metal deposition, additive manufacturing, process monitoring, coaxial optical monitoring, high-speed imaging, melt pool behavior, short-time correlation, intermittency

## Abstract

The development and improvement of monitoring and process control systems is one of the important ways of advancing laser metal deposition (LMD). The control of hydrodynamic, heat and mass transfer processes in LMD is extremely important, since these processes directly affect the crystallization of the melt and, accordingly, the microstructural properties and the overall quality of the synthesized part. In this article, the data of coaxial video monitoring of the LMD process were used to assess the features of melt dynamics. The obtained images were used to calculate the time dependences of the characteristics of the melt pool (MP) (temperature, width, length and area), which were further processed using the short-time correlation (STC) method. This approach made it possible to reveal local features of the joint behavior of the MP characteristics, and to analyze the nature of the melt dynamics. It was found that the behavior of the melt in the LMD is characterized by the presence of many time periods (patterns), during which it retains a certain ordered character. The features of behavior that are important from the point of view of process control systems design are noted. The approach used for the analysis of melt dynamics based on STC distributions of MP characteristics, as well as the method for determining the moments of pattern termination through the calculation of the correlation power, can be used in processing the results of online LMD diagnostics, as well as in process control systems.

## 1. Introduction

Laser metal deposition (LMD) is becoming an important component of modern digital manufacturing. This technology makes it possible to create metal products of complex shapes through the local interaction of concentrated flows of laser energy and powder material. In recent years, there has been an increase in the number of scientific publications devoted to various aspects of LMD, as well as an increase in the number of commercially available machines and equipment items that implement this technology. Nevertheless, the task of maintaining optimal process parameters that ensure the achievement of the desired physical and mechanical properties of the part is still a serious challenge. This is especially true for parts of complex geometry, including solid and thin-walled or overhanging elements, which predetermines variable heat transfer conditions along the construction path. In general, the situation is due to the general complexity of the technology—the presence of many mutually influencing physical processes accompanying additive manufacturing.

The Marangoni effect is typical for laser processing of metallic materials associated with the formation of the melt volume. It is caused by the surface tension gradient due to the inhomogeneous temperature field on the surface. As a result, thermocapillary forces arise at the free boundary, which bring the material in the molten pool (MP) into active motion, including gas phase transfer. The hydrodynamic processes occurring in the molten material largely predetermine the characteristic features of the technology under consideration. The dynamics of the melt and heat and mass transfer processes affect both the penetration depth and the final shape of the track, and the microstructural features of the material, and the formation of defects.

The processes of melt convection under the action of Marangoni forces have previously been studied in laser welding of metals. For example, in [1], the flow of a liquid in MP was studied using numerical simulation, and the temperature profile on its surface was determined taking into account the Marangoni effect. In [2], the influence of the Marangoni effect on the efficiency of convective heat transfer in MP was shown. In [3], the importance of taking into account thermocapillary convection when analyzing the formation of irregularities in the weld profile was shown. Thermocapillary instability and the development of convective flow under the action of Marangoni forces were previously analyzed in [4,5]. In [6], 3D simulation was used to study the processes of heat transfer and thermocapillary convection in LMD with coaxial powder injection. The formation of convection with a certain structure of vortex motion in MP is shown. In [7], the influence of the Marangoni effect and emerging convective flows on the formation of pores at LMD was analyzed. It is concluded that the number of defects depends on the process parameters and the dynamics of the molten pool. It was also shown [8] that convective flows in the melt, under certain conditions, reduce the porosity of the formed material.

Many physical phenomena are characterized by the so-called “intermittent behavior”, which occurs both in technical systems and in natural processes. Intermittency is the alternation of phases of regular and chaotic dynamics in nonlinear dissipative systems. Intermittent dynamics is one of the routes from regular dynamics to chaos in the parameter space of the dynamical system under considerations [9,10]. In hydrodynamics, this term denotes the appearance and dissipation of structural formations of various scales in a turbulent medium at sufficiently high Reynolds numbers [11]. Intermittency is also manifested in media with weak (wave) turbulence during Rayleigh–Bernard convection [12], Bernard–Marangoni [13], as well as in nonlinear dissipative systems described by the Kuramoto–Sivashinsky equation [14].

Thermocapillary convection is a special case of Benard–Marangoni convection, in which external factors set the temperature gradient on the liquid surface [15]. The route to chaos in the case of thermocapillary flow is described in [9,16]. The intermittency route in this case can be realized if additional conditions are met. Additional sources of motion are required, for example, concentration inhomogeneities [10], or it can be realized in systems with a low Prandtl number (*Pr*) [13], which is satisfied for metal melts (Pr≪1).

Currently, the phenomenon of intermittency in laser thermal metal processing technologies has not been sufficiently investigated. This is largely due to the limited possibilities of capturing the dynamics of the melt. The metal is opaque in the optical wavelength range, and convective flows can be detected only on the surface of the melt [13]. Regular periodic or quasiperiodic behavior in convective flow has been studied, for example, in laser welding [17,18] and in laser surface treatment [19]. It is indicated in [20] that the intensity of thermocapillary convection in LMD is variable under conditions of instability of the shape of the free surface, since the absorption of laser radiation is modulated by its relief.

One of the important directions of LMD development is the development and improvement of monitoring and management systems. The control of hydrodynamic and heat-mass transfer processes is extremely important, since they directly affect the processes of melt crystallization and, accordingly, the microstructural properties and quality of the synthesized part. An overview of diagnostic methods for the LMD process is presented in [21,22]. The analysis of video images of the zone of laser radiation exposure to the material is a relevant approach for monitoring and research of LMD. Researchers agree that the shape of MP and the temperature distribution on its surface carry information about the dynamics of the process [22,23]. The literature discusses both methods for determining MP characteristics from images and approaches to online correction of technological parameters based on the data obtained. In [24,25], MP coaxial video monitoring was used with automatic extraction of its characteristics from the obtained images in LMD. The dependences of the pool size on the number of layers at different technological parameters are presented. However, in these works, the average values for entire tracks or layers are investigated, and their temporal dynamics is not evaluated. The papers [26,27] show temporal or spatial dependences of MP sizes, but do not analyze the joint dynamics of several characteristics of the pool, which may be useful for obtaining additional information about the dynamics of the melt.

This article examines the dynamics of the melt at LMD based on the analysis of the sequence of images obtained using coaxial video monitoring. A set of key MP characteristics is calculated from the obtained images of the laser radiation exposure area. The obtained time dependences of the characteristics are analyzed using the Short-time correlation approach. This approach allows us to identify the features of the mutual dynamics of signals, as well as to analyze both local features of their behavior and changes in their dynamics by the type of intermittent behavior.

## 2. Materials and Methods

### 2.1. Experimental Setup

**Figure 1:**
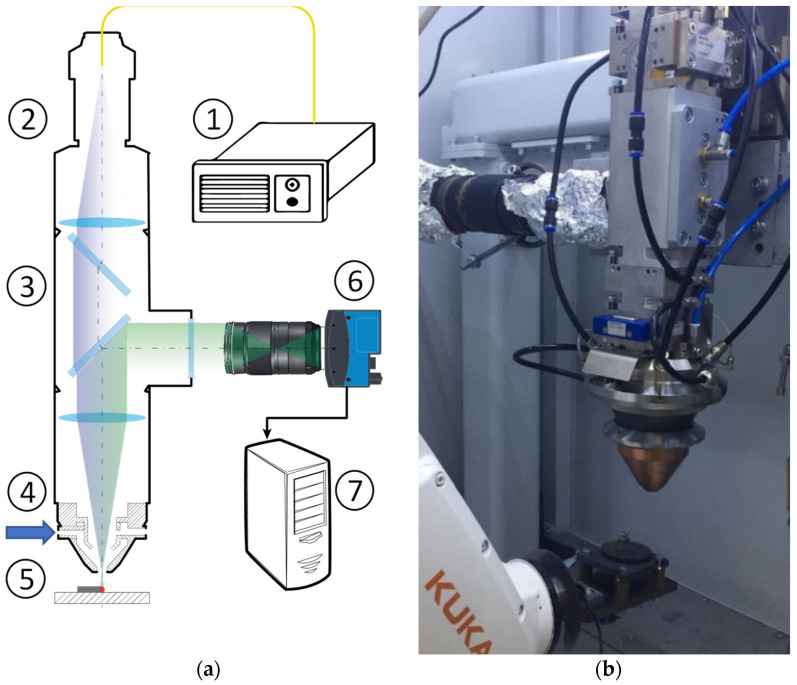
(**a**) Schematic view of the experimental setup: 1—laser; 2—laser head; 3—additional block of optical elements; 4—supply of gas-powder mixture; 5—substrate; 6—video camera; 7—frame grabber. (**b**) General view of the setup.

A research facility for laser metal deposition developed at ILIT RAS was used in this work. The setup schematic diagram is shown in Figure 1a. The setup uses a 400 W ytterbium fiber laser with a radiation wavelength of 1.07 μm (LK-400-V, NTO IRE-Polyus, Fryazino, Russia). The laser head (YC52, Precitec, Gaggenau, Germany) is fixed in an upright position. The substrate on the surface of which the material is deposited is driven by a 6-axis robotic arm (KR10 R900-2, KUKA, Augsburg, Germany). The focal length of the collimator lens and the focusing lens is the same and is 200 mm. The gas-powder mixture is fed by a powder feeder (PF 2/1LC, GTV, Luckenbach, Germany) through the coaxial nozzle of the laser head. The general view of the setup is shown in Figure 1b.

To diagnose the processes of heat and mass transfer in the MP accompanying the deposition of the track, an optical diagnostics (OD) complex has been developed. It is built into the optical path of the setup using a coaxial scheme. The OD uses a Mikrotron 3110 high-speed camera (Mikrotron, Unterschleissheim, Germany). The OD video subsystem captures the horizontal projection of the MP region. The total magnification of the system was ×1.4.

When using MP video diagnostics, there are a number of interfering factors, which include: Luminescent glow of oxides or slag on the MP surface, glow of plasma and metal vapors and reflection of laser radiation from the melt surface [22,28,29]. The use of selective optical filters makes it possible to exclude or reduce the influence of these factors. For the optical elements used in the developed OD, the required characteristics of spectral transmission and reflection were provided by using the technology of vacuum deposition of multilayer interference coatings [30]. The filter transmission in the range of 480–570 nm was more than 90%; in the rest of the visible range—less than 30%; at the radiation wavelength—less than 0.01%.

### 2.2. Image Processing

To determine the MP characteristics based on the data obtained in the process of video diagnostics, the developed image analysis algorithm is used. The algorithm detects the MP region on the images and calculates a set of key characteristics for it. When analyzing images, bright regions at the MP boundaries corresponding to areas with high radiation intensity are detected and excluded from consideration. Such areas appear to be oxidized solid material, or slag particles. Overestimated temperature values in such areas are due to a significantly higher emissivity of the material compared to the melt. Details of the algorithm implementation will be published in a separate article.

**Figure 2:**
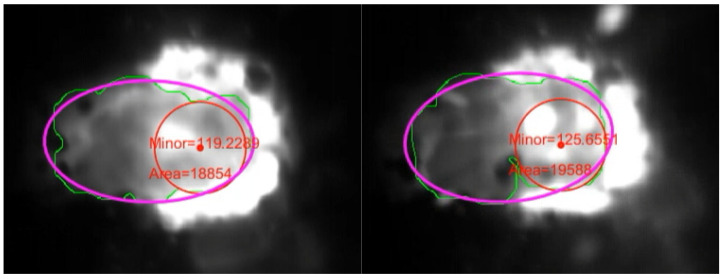
Coaxial camera images showing the melt pool area (green line), the equivalent ellipse (pink line) and the approximate position of the laser beam spot (red line).

After the MP region is selected, a so-called equivalent ellipse is determined for it, the lengths of the axes of which are further used to characterize the size of the pool. The algorithm allows real-time extraction of the following MP characteristics: Mean and standard deviation of pixel brightness values, integrated brightness, area and perimeter of the MP region, lengths of the minor (*Mi*) and major (*Mj*) axes of the equivalent ellipse. Herewith, the area and perimeter are determined for the actual detected region of the pool. An example of an image from a camera with the designation of the areas of the MP, the equivalent ellipse and the approximate position of the laser beam spot is shown in Figure 2.

For each frame, the pool surface temperature was also calculated. For this, the value of the average brightness in the MP zone was used, taking into account the transmission of the optical filter in the green region of the spectrum and the exposure time [31]. The calibration of the brightness pyrometry method was carried out according to the known melting temperature at the crystallization boundary. The temperature measurement range was adjusted by changing the exposure time. Estimates have shown that variation of the emissivity with a change in the phase state of the material leads to an error in determining the temperature of less than 3%.

### 2.3. Experimental Parameters

During the experimental work, a series of single track depositions was formed using LMD. At the same time, video capturing of MP dynamics was performed using a high-speed OD camera. The length of the tracks was 30 mm. The power used was 320 W, the scanning velocity $V_{s}$ took values of 5, 8, 10 mm/s. The distance from the focus of the beam to the substrate was 9 mm. In the plane of the substrate, the beam had a Gaussian intensity distribution, its characteristic size at the intensity level $1/e^{2}$ was: $D_{b}$ ≈ 1.26 mm. AISI 304 austenitic steel powder with a grain size distribution of 40–100 μm was used, the powder mass flow rate was 0.14 g/s.

Images of the MP surface were captured using 2800 Hz frequency with a frame size of 560 × 280 pixels and an exposure time of 0.3 ms. The spatial resolution was about 5.7 µm/pixel. As a result of processing the sequence of pool images, the time dependences of MP characteristics were obtained.

### 2.4. Short Time Correlation

The short-time correlation (STC) method was used to analyze the dynamics of the MP characteristics, as well as their relationship. This method makes it possible to investigate the correlations of a set of consecutive short samples from the analyzed signals. As a result, it is possible, within short times, to isolate interconnections in signals, estimate the rate of change of signals and detect oscillatory components against the noise and interference [32,33]. The principle of constructing this transformation is close to the short-time Fourier transform [34] and wavelet transform [35], which were previously used to analyze intermittent dynamics and the transition to chaos. The original signals are divided into overlapping samples of length N, after which the correlation functions are calculated for the corresponding samples:

(1) $$r\left(t_{k},l\right)=\overset{N-1}{\underset{m=0}{\sum }}f\left(t_{k}+m\right)\cdot g\left(t_{k}+m+l\right)$$

where −N<l<N.

The resulting correlograms are displayed in vertical columns on the final STC distribution. To form the *k*-th column, which corresponds to the time $t_{k}$, the *k*-th correlogram is used. The resulting 2D STC distributions $r\left(t_{k},l\right)$ display the value of the correlation coefficient depending on the time $t_{k}$, starting from which a short sample is taken from the signals, as well as the correlation lag l. The brightness of the points of the STC distribution is proportional to the amplitude of the correlogram, and the color is proportional to the sign: Red indicates a positive correlation, blue indicates a negative (anticorrelation). STC distributions are sensitive to oscillatory processes: If there are periodic changes in the local region, they repeat from correlogram to correlogram, and a set of stripes parallel to the abscissa axis is observed on the STC distribution in this region. The step of the stripes allows you to estimate the repetition period. In the future, to indicate the STC distribution for two different signals *S1* and *S2* (cross-correlation), we will use the notation *<S1-S2>*, whereas the STC distribution of the signal *S1* with itself (autocorrelation) will be denoted as *<S1-S1>*.

We used the duration of short samples equal to the time it takes for the beam to travel the characteristic MP scale. Since the velocities of convective motion and propagation of disturbances over the surface exceed the scanning velocity, then the characteristic times of change of the analyzed signals associated with these processes are within the considered interval. As a characteristic scale for MP, we used its width, which for our experimental conditions can be considered equal to D≈1 mm. The resulting scale of STC distributions along the ordinate axis is ±D≈2 mm, and along the abscissa axis it corresponds to a track length of 30 mm.

Before calculating the STC distributions, the received signals were subjected to median filtering and interpolation. Note that the STC transform suppresses the noise component of the signal depending on the sample length. The superposition of a set of incoherent oscillations, the scale of which is much smaller than the sample length, does not affect the final value of the correlation. The final spatial step of signal sampling, taking into account the scanning velocity, was 24 μm.

## 3. Results and Discussion

### 3.1. Experimental Results

**Figure 3:**
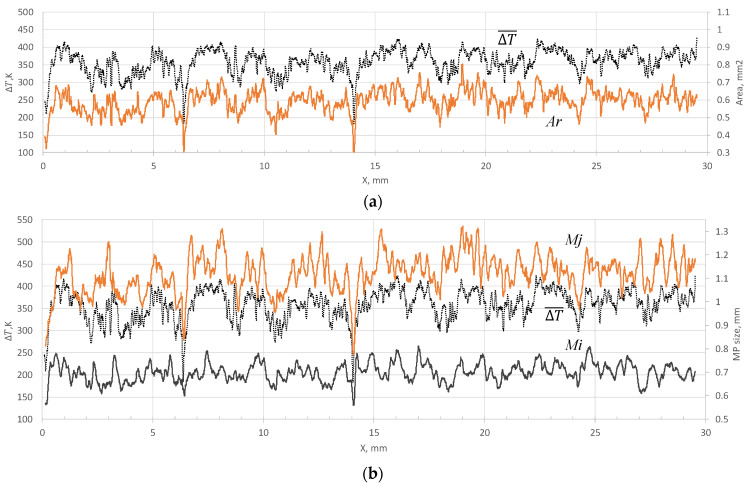
Spatial dependences of the signals received in the regime *P* = 320 W, $V_{s}\;$= 10 mm/s. (**a**) Inverted temperature ($\bar{∆T}$ ) and pool area (*Ar*), (**b**) length (*Mj*), width (*Mi*) of the pool and ($\bar{∆T}$ ).

Typical spatial dependences of the MP characteristics are shown in Figure 3. The spatial coordinate is calculated as $x_{k}=V_{s}t_{k}$. Figure 3a shows the dependencies of the MP area (*Ar*) and $\bar{∆T}$, and Figure 3b shows the length (*Mj*) and width (*Mi*) of the pool, as well as $\bar{∆T}$. Here $\bar{∆T}=\left(2700\;K-T\right)$ is an inverted value introduced for the convenience of comparing the dynamics of temperature (*T*) and the size of the MP. There is a pronounced negative correlation between the pool area and the temperature on its surface: With an increase in temperature, the melt area decreases, and vice versa—an increase in the melt area corresponds to the drop of the temperature. The relationship between temperature and the values of *Mi*, *Mj* values is much more complicated, although these values are also dimensional characteristics of the MP. Changes in *Mi* and *Mj* can both occur simultaneously or be uncorrelated. In general, it is quite difficult to assess the evolution of their relationship by time (or spatial) dependencies. It is necessary to use a different approach to analyze their joint dynamics.

**Figure 4:**
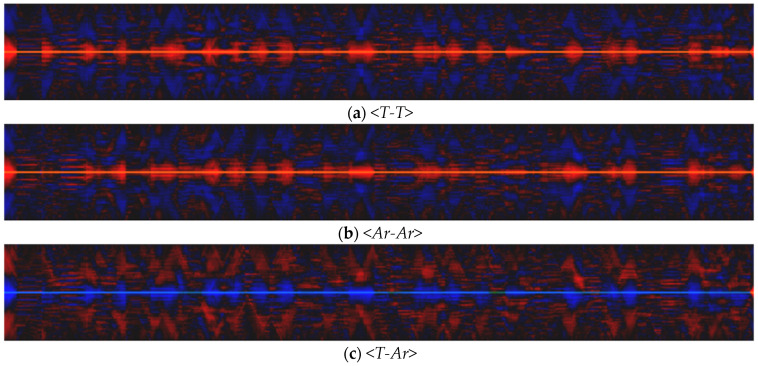
STC distributions obtained in the regime *P* = 320 W, $V_{s}\;$= 10 mm/s: (**a**) <*T-T*>; (**b**) <*Ar-Ar*>; (**c**) <*T-Ar*>; red color indicates a positive correlation, blue color indicates a negative one.

**Figure 5:**
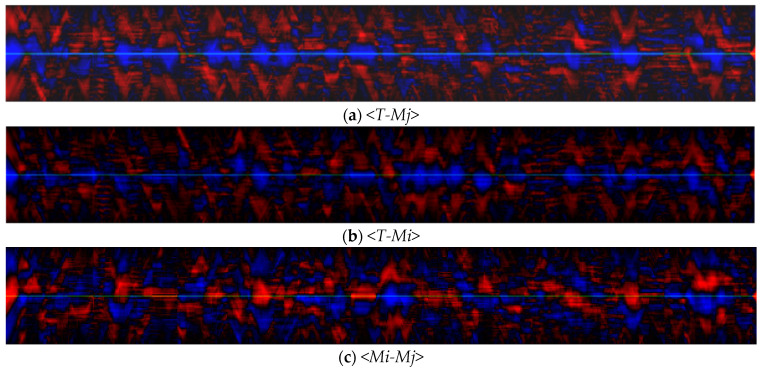
STC distributions, using the length and width of the pool (*P* = 320 W, $V_{s}\;$= 10 mm/s): (**a**) <*T-Mj*>; (**b**) <*T-Mi*>; (**c**) <*Mi-Mj*>; red color indicates a positive correlation; blue color indicates a negative one.

Variations in the width of the pool usually do not exceed 200 µm, i.e., changes occur on the scale of several particle diameters. The length of the pool can vary significantly more. Depending on the regime, the ratio of the maximum length value to the minimum can reach 1.5. When analyzing the dynamics of signals using the STC approach, the main factors affecting the distributions are the mutual behavior of signals, as well as the relative scale of their changes within the sample. Figure 4 and Figure 5 shows STC distributions for a scanning velocity of 10 mm/s. Analysis of the distributions obtained for all the scanning velocities used shows that their structure is similar. In the structure of the STC distributions *<T-T>* and *<Ar-Ar>* (Figure 4a,b) there are many stripes parallel to the abscissa axis.

This type of distribution displays the oscillatory changes of the corresponding signals. Parallel stripes are also observed on the joint STC distribution *<T-Ar>* (Figure 4c), which indicates the consistent nature of changes in temperature and the area of the MP at this scale. The spatial oscillation period is in the range of 0.03–0.07 mm and corresponds to frequencies of about 140–300 Hz. It is important to note that the period of variation of the quantities is much smaller than the size of the laser spot: $V_{s}/F<D_{b}/6$, where *F* is the frequency observed in the experiment. There are periods of pronounced anticorrelation of values on the *<T–Ar>* distribution, which corresponds to a decrease in temperature with an increase in the melt area or vice versa. This behavior occurs on a much larger spatial scale of 0.5–2 mm and does not have a pronounced oscillatory character.

STC distributions, including the length and width of the pool (Figure 5), have a different structure compared to the distributions constructed using the area signal. Their joint STC distribution *<Mi-Mj>* (Figure 5c) consists of separate periods of positive and negative correlation with a length of 0.5–2 mm, as well as transitions between them. The high-frequency periodic component is also determined on the distributions. Its spatial scales correspond to temperature and area fluctuations and are 0.03–0.07 mm. Thus, the spatial and temporal scales of changes in the length and width of the pool are close to each other; however, they are not always consistent, and the trends of their changes can both coincide and be opposite.

The interrelation of the distributions *<Mi-Mj>* and *<T-Ar>* is interesting—during the periods when a positive relationship between the length and width of the pool is observed, the anticorrelation of the signals of the temperature and pool area is recorded. A negative relationship is also recorded in separate distributions *<T-Mj>* and *<T-Mi>*. At the same time, during periods when the relationship between the length and width is negative, the dynamics of *<Ar-T>* is generally independent and individual relationships *<T-Mj>* and *<T-Mi>* are multidirectional or absent. Since, by definition, Ar~Mj·Mi, it can be assumed that the dynamics of the length and width of the pool are simultaneously influenced by various processes, some of which do not change the area of the pool as a whole. This is important to take into account when designing monitoring and control systems for the LMD process, since controlling the melt volume by tracking only the width or only the length of the pool may be less effective than tracking its area.

Thus, in the analyzed signals, it is possible to identify moments of time at which a rather sharp change in the nature of their dynamics occurs. Such changes occur consistently in all signals and are probably a reflection of a single process in MP. Between such moments of time, the nature of the dynamics of the signals changes insignificantly. As a result, the distributions are divided along the abscissa axis into separate sections of a similar structure (hereinafter—patterns) with a duration of 50–200 ms (5–20 Hz) and a length of 0.5–2 mm. The moments of pattern change on STC distributions correspond to areas of reduced brightness. Since the brightness on the distributions is proportional to the magnitude of the correlation function, the time intervals of reduced brightness between patterns indicate a local decrease in the interconnection of signals.

### 3.2. Measure of Relationship

To assess the local degree of ordering of the processes, we calculate the distributions of the correlation power according to STC data. For each coordinate, we sum up the squares of the values of the correlation coefficient over the entire correlogram:

(2) $$E\left(m\right)=\underset{l}{\sum }r{\left(m,l\right)}^{2}$$

**Figure 6:**
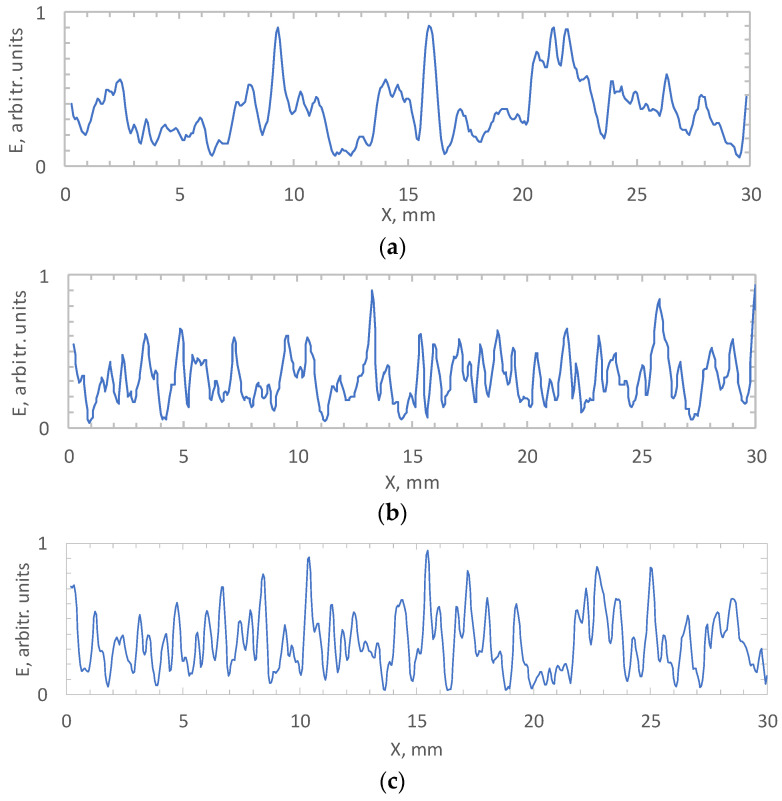
Normalized power distributions of the STC <*Mi-Mj*> for three values of the scanning velocity: (**a**) 5 mm/s; (**b**) 8 mm/s; (**c**) 10 mm/s.

This value reflects the measure of interconnection, ordering and regularity of signals on the local interval ±D. Figure 6 shows the normalized power distributions of the STC *<Mi-Mj>*, for three values of the scanning velocity. We use the *<Mi-Mj>* distribution, since it contains the highest correlation values, which gives a more contrasting dependence E(m). Nevertheless, using other distributions gives a qualitatively similar result. The resulting correlation power dependence has many local minima that reflect the moments of pattern change. The function passes through a minimum when the relationship in the analyzed signals disappears, the structure of fluctuations changes or the trend changes. Table 1 shows the number of local minima, as well as the average values of the duration and spatial size of the patterns. Note that the average length of the pattern is less than $D_{b}$ and decreases with increasing velocity.

### 3.3. Signal Interpretation

We associate the observed fluctuations in temperature and pool sizes at frequencies of 140–300 Hz with the convective motion of the melt. The formation of vortex flows is caused by thermocapillary forces arising as a result of high temperature gradients on the surface [4,36]. In the process of convective mixing, heated melt masses are submerged downward, and cold masses are carried upward, which leads to periodic changes in the surface temperature. Temperature fluctuations can also be associated with the displacement of temperature inhomogeneities caused by nonuniform absorption of laser radiation on variations in surface relief [37]. The intense movement of the melt also leads to periodic displacement of the free boundaries of the pool with the rotation frequency of the vortex, which is displayed by fluctuations in the pool size signals.

Thus, the fine structure of STC distributions reflects the internal structure of the convective flow. Since such fluctuations in the temperature and pool size signals are caused by the same processes, the signal changes occur in a correlated manner. Within individual patterns, the distribution structure is regular, which corresponds to the preservation of the spatial structure of the vortex motion.

Slow changes in the dimensions of the pool at frequencies of 5–20 Hz are caused by large-scale processes associated with a general change in the geometry of the pool, inertial motion of large volumes of the melt, as well as processes involving a general increase or decrease in temperature in the pool, restructuring of convective structures with a change in the overall efficiency of heat transfer. The shape of MP can change under the action of the emerging flows caused by thermocapillary forces and during relaxation oscillations of the free boundaries of the pool.

The structure of vortex flows inside the pool (the number and configuration of vortices, their speed) is determined by a combination of external factors—the amplitude and direction of thermocapillary forces, the volume and shape of the pool. The problem of realizability of the vortex motion structure under Benard–Marangoni convection was considered in [38], where the dependence of the flow configuration on the conditions of convection development was shown. At each cycle of the development of a convective flow, vortex structures appear, which can either repeat the previous configuration of flows or form a new one. The formed structure remains stable for some time until the external conditions change sufficiently. When the external conditions change, the vortex structure adapts. In this case, the level of short time correlation drops and a decrease in brightness is observed on the STC distributions.

In the case of a decrease in the scale of thermocapillary forces, viscous forces redistribute the movement in the pool, the motion loses ordering and the regularity in the obtained signals decreases. The absence of a pronounced regular component and the independence of signal changes are also displayed on STC distributions by periods of reduced brightness.

### 3.4. Melt Pool Dynamics Evaluation

Let us estimate the parameters of the dynamics of the melt corresponding to the observed experimental data. Let us assume that the structure of convection developed under the action of Marangoni forces consists of a toroidal vortex [6] with external dimensions $D_{T}$ slightly less than *D*. Let us take $D_{T}\approx 0.8\;\mathrm{mm}$, and use the rotation frequency F≈200 Hz. In this case, the maximum speed of movement of the melt is *U*:

(3) U=F·2πDT/4≈0.25 ms

Similar values of the vortex motion velocity were obtained earlier in calculations of the effect of laser radiation on metal during welding, taking into account Marangoni convection [2], and in the calculations of melt motion in LMD technology at certain technological parameters [39]. Similar values were also obtained by X-ray analysis of the velocity of gas bubbles in the melt in LMD [40].

The pool is also exposed to falling particles. The collision frequency can be estimated as $f_{p}=\dot{m}k_{e}/m_{p}$, where $\dot{m}$ is powder mass flow rate, $k_{e}$ is the particle capture efficiency, $m_{p}$ is the mass of one particle. For the parameters used in the experiment, the collision frequencies are $f_{p}>25\;\mathrm{kHz}$. Under the impact of powder particles, the surface of the pool is curved. The deformations induced in this way form capillary waves propagating over the surface of the pool. The velocity of capillary waves can be estimated as $V_{cap}=\sqrt{\frac{2\pi \sigma }{\rho \cdot \lambda }}$, where σ is the surface tension, λ is the length of the capillary wave. Waves with a wavelength greater than the size of the pool are not realized. The phase velocity of the capillary wave with a length less than the size of the pool is $V_{cap}>1.5\;m/s$. Thus, the diagnostic regimes used do not allow us to observe capillary waves, since in one sampling step, they propagate over a distance greater than the size of MP.

The temperature difference between the heated surface and the lower boundary of the pool can be estimated from a two-dimensional model of laser heating of the MP surface taking into account convection [41]. Taking into account the found vortex rotation frequency and the taken estimate of the vortex radius, we obtain:

(4) ∆T=ε·Pρ·Cp·π2·F·DT316

where ε=0.5 is the efficiency of laser heating of the surface, *P* is the power of laser radiation, $\rho \approx 7900\mathrm{kg}/m^{3}$ is the density of steel, $C_{p}\approx 502J/\mathrm{kg}\cdot K$ is the specific heat. Substituting the values of the quantities in (4) we obtain ∆T≈650 K. Taking into account that the temperature at the boundary of the phase transition for stainless steel is $T_{m}\approx 1710\;K$, we obtain an estimate of the temperature at the surface T≈2360 K. Previously, we [42] experimentally investigated the average surface temperature in LMD using a pyrometer of spectral ratio. For the experimental conditions corresponding to this article, an average surface temperature of 2300 K was obtained, which is close to the estimated value obtained.

### 3.5. Intermittent Behavior

The experimental data obtained indicate that the behavior of the melt in the LMD process is characterized by the presence of many time periods, during which a certain ordered character is preserved in it. During the transition between such periods, the dynamics of the melt loses ordering for a certain time. At the same time, in different periods, a different structure of movement can be realized. Such behavior of the melt with alternating phases of regular dynamics and moments of reduced ordering can be interpreted as a manifestation of the intermittent behavior of the melt.

The efficiency of convective heat transfer from the surface to the depth of the pool may differ for different vortex configurations. Special thermodynamic conditions will be established in different patterns, which, following the intermittent dynamics of the melt, will cause periodic changes in the efficiency of heat and mass transfer. Such changes can potentially affect both the local penetration depth and the shape of the track. In this case, the melt dynamics analysis method considered in the article based on STC distributions of MP characteristics, as well as an approach for determining the moments of pattern termination through the calculation of correlation power, can be used in the processing of monitoring data and in LMD process control systems.

### 3.6. Periodic Dynamics

A special regime of large-scale evolution of the MP, which can be realized in the LMD, includes the cyclic dynamics of the birth-decay of convective processes. Such a regime describes well the observed phenomena, including the intermittent behavior of the melt; however, in our opinion, it is not necessarily realized in every pattern. At the initial moment, as a result of local absorption of laser energy, the temperature on the surface begins to rise. Temperature gradients increase, and the emerging Marangoni forces set the melt in motion. Convective mixing of the melt arises and intensifies, increasing the efficiency of heat transfer from the surface of the pool to the depth. The phase boundary at the bottom of the pool moves towards the solid material, the volume of the pool and its surface area increase. Active mixing of the melt causes periodic deformation of the surface, as well as the formation and movement of temperature inhomogeneities over the surface, which are recorded as the temperature fluctuations discussed above. The nature of the dynamics of the temperature signals and pool sizes at this stage is determined by the structure of the convective flows of the melt. As the excess heat is distributed throughout the pool, and the temperature inhomogeneities on the surface are leveled out, the source of motion—the Marangoni forces—weakens. Viscous forces begin to prevail, the vortex motion slows down and the efficiency of heat transfer to the depth of the MP decreases. With a decrease in temperature in the phase transition region, its boundary begins to shift towards the melt, the volume and area of the pool decrease. The nature of the convective dynamics of the melt changes, which also changes the structure of the obtained signals. A decrease in the regularity of signals dynamics is reflected in the STC distributions by the end of the pattern. As laser energy continues to be absorbed, a decrease in surface cooling efficiency leads to a new heating cycle and convective motion, as well as a new pattern in the STC distribution.

It is important to emphasize the decisive influence of convection in the melt on the transfer of heat from the surface to the inner boundaries of the MP. In our opinion, it is the dependence of the heat transfer efficiency on the nature of convection that predetermines the periodic dynamics in the pool observed in the experiment. The consequence of this is the periodic appearance of multidirectional dynamics of temperature and melt area signals. The reduction of the pool area occurs when heat transfer to the phase boundary is not efficient enough. As a result, the volume of the pool is reduced, and the incoming laser energy leads to a significant rise in the surface temperature, up to overheating. An increase in the volume and area of the pool occurs as the efficiency of heat transfer to the depth of the pool is restored. In this case, the surface temperature decreases. This feature of signal dynamics must be taken into account when designing monitoring and control systems for the LMD process. Periodic changes in the temperature signals and pool dimensions with a lag relative to each other, with incorrect tuning of the control systems, can lead to an increase in the amplitude of oscillations and the exit of the control object from the equilibrium state.

One more remark can be made in connection with the observed periodic changes in the MP length. Since the front boundary of the pool is set by the position of the laser spot, the observed fluctuations in the pool length are provided by the movement of the rear edge relative to the beam. Thus, the crystallization process at the trailing MP edge occurs at a variable rate, periodically slowing down and accelerating at a constant scanning velocity. Such periodicity is inevitably reflected in the microstructural properties of the solidified material.

## 4. Conclusions

In this article, a study of the dynamics of the melt during LMD is carried out according to the data of coaxial video monitoring of the area of exposure to laser radiation. The obtained images were used to calculate the time dependences of the key characteristics of the melt pool (temperature, width, length and area), which were further processed using the short-time correlation (STC) method. This approach made it possible to reveal the local features of the joint behavior of the MP characteristics, and due to this, to analyze the nature of the dynamics of the melt and the features of its evolution.

The experimental data obtained show that the behavior of the melt in the LMD process is characterized by the presence of many time periods (patterns), during which a certain ordered character is preserved in it. During the transition between such periods, the dynamics of the melt loses ordering for a certain time. At the same time, in different periods, a different structure of movement can be realized. Such behavior of the melt with alternating phases of regular dynamics and moments of reduced ordering can be interpreted as a manifestation of the intermittent behavior of the melt. The end points of the patterns can be determined, also in automatic mode, by calculating the power of the correlation. Since the efficiency of convective heat transfer in the pool for different vortex configurations may be different, periodic changes in the penetration depth and the shape of the track may occur following the intermittent dynamics of the melt.

Regarding the design aspects of monitoring and control systems for the LMD process, the article notes that the pool length and width signals exhibit complex behavior, and are determined, apparently, simultaneously by different processes. In this regard, monitoring the volume of the melt by tracking only the width or only the length of the pool may be less effective than tracking its area. Moreover, in order to avoid an increase in the amplitude of oscillations, it is necessary to take into account the periodic changes in the signals of the temperature and the dimensions of the pool, which occur with a delay relative to each other.

We believe that the approach used in the article to analyze the dynamics of the melt based on STC distributions of MP characteristics, as well as the method for determining the moments of pattern termination through the calculation of the correlation power, can be used in the interpretation of the results of online diagnostics of the LMD, as well as in process control systems.

## Figures and Tables

**Table 1:** Pattern characteristics determined by normalized power distributions of the STC <*Mi-Mj*>.

Scanning velocity, mm/s	5	8	10
Number of local minimums, pcs	34	41	43
Average pattern duration, ms	176	91	70
Average pattern length, mm	0.88	0.73	0.7

## Data Availability

The data presented in this study are available on request from the corresponding author.
